# Association of *CYP2C9∗3* and *CYP2C8∗3* Non-Functional Alleles with Ibuprofen-Induced Upper Gastrointestinal Toxicity in a Saudi Patient

**DOI:** 10.1155/2023/6623269

**Published:** 2023-07-21

**Authors:** Amina M. Bagher

**Affiliations:** Department of Pharmacology and Toxicology, Faculty of Pharmacy, King Abdulaziz University, Jeddah, Saudi Arabia

## Abstract

Ibuprofen is a non-steroidal anti-inflammatory drug (NSAID) widely used to alleviate pain and inflammation. Although it is generally considered safe, common adverse drug reactions of ibuprofen include stomach pain, nausea, and heartburn. It can also cause gastrointestinal (GI) bleeding, especially in individuals with a history of GI ulcers or bleeding disorders. Ibuprofen is predominantly metabolized by the cytochrome P450 (CYP) enzymes CYP2C9 and CYP2C8. Individuals carrying the *CYP2C9*∗*3* or *CYP2C8*∗*3* non-functional alleles have reduced enzyme activities resulting in elevated ibuprofen plasma concentrations and half-life. We presented a case of a 31-year-old Saudi female patient with a history of rheumatoid arthritis (RA) who had taken ibuprofen at 600 mg twice daily for eight weeks. The patient presented to the emergency department with symptoms including nausea, vomiting, severe abdominal pain, and black tarry stools. An emergency esophagogastroduodenoscopy was performed on the patient, which revealed a deep bleeding ulcer measuring 1 × 1 cm in the antrum of the stomach. Laboratory investigations indicated anemia (hemoglobin: 7.21 g/dL and hematocrit: 22.40 g/dl). The patient received intravenous proton pump inhibitors and a packed red blood cell transfusion. Genetic analysis revealed that the patient was a carrier of *CYP2C9*∗*3 and CYP2C8*∗*3* variant alleles, indicating that the patient is a poor metabolizer for both enzymes. The patient's symptoms improved over the subsequent days, and she was discharged with instructions to avoid NSAIDs. This is the first reported Saudi patient homozygous for *CYP2C9*∗*3 and CYP2C8*∗*3* variant alleles, which led to ibuprofen-induced upper GI toxicity. This case demonstrates the importance of contemplating *CYP2C9* and CYP2C8 genetic variations when administrating NSAIDs like ibuprofen. Careful assessment of the risks and benefits of NSAID therapy in each patient and consideration of alternative pain management strategies must be conducted when appropriate.

## 1. Introduction

Ibuprofen is a non-steroidal anti-inflammatory agent (NSAID) commonly used to alleviate moderate pain and fever and to treat rheumatoid arthritis, osteoarthritis, and ankylosing spondylitis [1, 2]. Ibuprofen is a cyclooxygenase-1 (COX-1) and COX-2 inhibitor, catalyzing prostaglandin synthesis. The inhibition of COX-1 and COX-2 may account for ibuprofen's antipyretic, analgesic, and anti-inflammatory effects [3, 4]. Ibuprofen is available over the counter in most countries and is generally considered safe. However, like other NSAIDs, ibuprofen can cause potential adverse drug reactions (ADRs), which include nausea, vomiting, gastrointestinal (GI) discomfort, diarrhea, and headache [5–7]. Ibuprofen can also cause serious complications, including GI bleeding and ulcers (1-2%), hypertension (up to 5%), myocardial infarction (up to 1% per year), and heart failure (up to 1%), and renal damage and sudden cardiac death have also been observed in rare cases [8].

Ibuprofen is predominantly metabolized by the cytochrome P450 (CYP) enzymes CYP2C9 and CYP2C8 to form the inactive metabolites hydroxyibuprofen and carboxyibuprofen [9, 10]. These metabolites are then eliminated from the body through the urine. The rate of metabolism and elimination of ibuprofen can vary depending on factors such as age, liver function, genetic variations in CYP enzymes, and concurrent use of other medications that affect CYP activity [9, 10].

Genetic polymorphisms in *CYP2C9* and *CYP2C8* genes can alter these enzymes' activity and the ibuprofen's metabolism, leading to variations in drug efficacy and ADRs [11–13]. The most studied *CYP2C9* polymorphisms are *CYP2C9*∗*2* (rs1799853) and *CYP2C9*∗*3* (rs1057910). Individuals carrying the *CYP2C9*∗*2* and *CYP2C9*∗*3* variant alleles have decreased enzymatic activities, approximately 5% and 12%, respectively, compared to individuals with the *CYP2C9*∗*1* wild-type allele [14]. As a result, individuals carrying these variant alleles may have slower metabolism of ibuprofen, leading to higher drug concentrations and a longer duration of action [15, 16]. Studies have shown that individuals carrying the *CYP2C9*∗*2 and CYP2C9*∗*3* variant alleles are at higher risk of developing ADRs and toxicities to ibuprofen, such as GI bleeding or kidney damage, compared to individuals carrying the wild-type *CYP2C9*∗*1* allele [17, 18]. Furthermore, individuals who carry the *CYP2C8*∗*3* (rs10509681) allele have reduced enzymatic activity relative to the wild-type *CYP2C8*∗*1* allele. The *CYP2C8*∗*3* allele has been linked to diminished ibuprofen metabolism and clearance [12, 19].

This paper presents a unique case involving a Saudi patient homozygous for both *CYP2C9*∗*3* and *CYP2C8*∗*3* variant alleles, which led to ibuprofen-induced upper GI toxicity. This is the first documented case reporting a combination of genetic variants and their association with ADRs following ibuprofen administration.

## 2. Case Presentation

We report a case of a 31-year-old Saudi female patient diagnosed with rheumatoid arthritis (RA) for one year. As part of her RA management, the patient has been prescribed a weekly oral dose of methotrexate at 7.5 mg and a daily folic acid supplementation of 1 mg/day for 12 months. Additionally, for the past eight weeks, the patient has been self-medicating with ibuprofen at 600 mg twice daily to alleviate acute pain associated with RA. The patient presented to the emergency department with symptoms including nausea, vomiting, severe abdominal pain, and black tarry stools. Notably, the patient reported that these symptoms started on the day she sought emergency services. The patient did not report any preexisting medical conditions, including GI disorders or ulcers. Furthermore, the patient had no history of smoking or alcohol consumption and exhibited a sedentary lifestyle.

On physical examination, the patient exhibited signs of hemodynamic instability. In the supine position, the patient's blood pressure was measured at 90/30 mmHg, indicating hypotension. Additionally, the patient displayed tachycardia, with a heart rate of 130 beats per minute. The abdominal examination demonstrated epigastric tenderness along with abdominal guarding and rigidity. Laboratory investigations showed anemia (hemoglobin: 7.21 g/dL (normal: 14–18 g/dl) and hematocrit: 22.40 g/dl (normal: 14–18 g/dl)) and mild leukocytosis (white blood cells (WBC): 13700/mm^3^ (normal: 4–11 10^3^/mm^3^)). Liver functions, blood sugar, urea, bilirubin, and coagulation parameters were all within normal ranges.

An emergency esophagogastroduodenoscopy (EGD) was performed on the patient, which revealed a deep ulcer measuring 1 × 1 cm in the antrum of the stomach. The ulcer was characterized by the presence of a visible vessel, consistent with a Forrest Ib classification, indicating active bleeding. Additionally, according to the Sakita classification, the ulcer was classified as Sakita Type II, representing a deep ulcer with a visible vessel or clot. Notably, the EGD examination did not reveal any additional abnormal endoscopic findings throughout the esophagus, duodenum, or other regions of the upper GI tract. Importantly, gastric biopsies obtained from both the antrum and body regions yielded negative results for *Helicobacter pylori* infection. The patient was diagnosed with a peptic ulcer and was promptly administered an intravenous proton pump inhibitor (PPI) alongside a transfusion of packed red blood cells to correct the accompanying anemia. The patient was hospitalized to closely monitor for any indications of recurrent bleeding.

**Figure 1:**
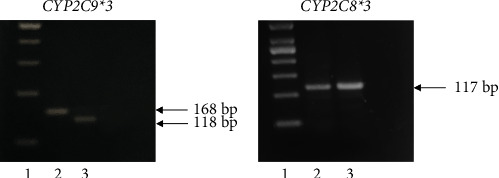
Polymerase chain reaction-restriction fragment length polymorphism (PCR-RFLP) results of *CYP2C9*∗*3* and *CYP2C8*∗*3* polymorphisms. (a) Agarose gel electrophoresis analysis for *CYP2C9*∗*3* polymorphism following digestion with *Mph1103I (NsiI)*. Lane 1: 100 bp DNA marker. Lane 2: undigested PCR product (168 bp). Lane 3: digestion of the homozygous mutant CC genotype yields two bands (50 and 118 bp). Since the molecular weight of 50 bp is very small, it could not be distinguished on the gel. The major visible band was of 118 bp. (b) Agarose gel electrophoresis analysis for *CYP2C8*∗*3* polymorphism following digestion with *PdmI (XmnI)*. Lane 1: 50 bp DNA marker. Lane 2: undigested PCR product (117 bp). Lane 3: digestion of the homozygous mutant GG genotype is not cut and yields one band (117 bp).

Analysis of genetic variations in *CYP2C9*∗*3* and *CYP2C8*∗*3*, which play a significant role in the metabolism of ibuprofen, was performed. The patient's DNA was collected from saliva samples using an Oragene DNA saliva collection kit (OG-500, Genotek, Canada), and genotyping of *CYP2C9*∗3 and *CYP2C8*∗3 alleles was performed using polymerase chain reaction-restriction fragment length polymorphism (PCR-RFLP) following previously described protocols [20, 21]. In brief, *CYP2C9*∗*3* was detected using the forward and reverse PCR primers 5′-TGCACGAGGTCCAGAGATGC-3′ and 5′-GATACTATGAATTTGGGGACTTC-3′ (Macrogen, Korea), respectively. The amplified PCR products were digested with 5 U restriction enzymes *Mph1103I (NsiI)* (Thermo Fisher Scientific, USA) overnight at 37°C. The detection for *CYP2C9*∗*3* was performed using the forward and reverse primers 5′-CTTCCGTGCTACATGATGACG-3′ and 5′-CTGCTGAGAAAGGCATGAAG-3′ (Macrogen, Korea), respectively. The amplified PCR products were digested with 5 U restriction enzymes *PdmI (XmnI)* (Thermo Fisher Scientific, USA) by overnight incubation at 55°C. The digested PCR products were separated by agarose gel electrophoresis and visualized under ultraviolet light. The representative agarose gel images of *CYP2C9*∗3 and *CYP2C8*∗2 PCR-RFLP assays were used to interpret the patient's genotypes (Figure 1). The *CYP2C9*∗3 allele produced 50 bp and 118 bp after digestion with *Mph1103I (NsiI)* (Figure 1(a)), while the *CYP2C8*∗*3* allele produced as a single band at 117 bp that is not cut following the digestion with *PdmI (XmnI)* (Figure 1(b)). The genotyping results revealed that the patient was homozygous for the *CYP2C9*∗*3* non-functional allele (*CYP2C9*∗*3/*∗*3*) and *CYP2C8*∗*3* non-functional allele (*CYP2C8*∗*3/*∗*3*). According to the Clinical Pharmacogenetics Implementation Consortium (CPIC), the patient is a CYP2C9 poor metabolizer (homozygous *CYP2C9*∗*3*) and CYP2C8 poor metabolizer (homozygous *CYP2C8*∗*3*). Poor metabolizers of CYP2C9 and CYP2C8 enzymes exhibit reduced metabolic activity, resulting in a diminished ability to metabolize ibuprofen. Consequently, these individuals experience slower clearance of the drug from their bodies. These genetic variations may have contributed to the patient's increased risk of GI ulcers while taking ibuprofen.

In the following days, there was a notable improvement in the patient's clinical condition. A repeat EGD was performed, revealing promising signs of ulcer healing. Consequently, the revised Forrest classification for the ulcer was determined to be III, indicating a healing ulcer. Correspondingly, the Sakita classification for the patient was categorized as Sakita Type III, providing further affirmation of the ongoing healing process of the ulcer. During the patient's hospitalization, a transition was made from intravenous to oral PPI therapy. After two days without any signs of rebleeding and with the patient's vital signs returning to normal, she was discharged from the hospital.

The patient's discharge plan involved maintaining the ongoing oral PPI treatment while discontinuing ibuprofen. Additionally, the patient received instructions to refrain from using NSAIDs and to schedule a follow-up appointment with her rheumatologist for further management of her RA.

## 3. Discussion

Ibuprofen is widely used to alleviate pain, inflammation, and fever. Although it is generally considered safe, prolonged or excessive use of ibuprofen can lead to various ADRs. Common ADRs of ibuprofen include stomach pain, nausea, and heartburn. It can also cause GI ulcers, especially in individuals with a history of GI ulcers or bleeding disorders [3, 22]. Specific polymorphisms in the genes encoding the CYP2C9 and CYP2C8 enzymes can lead to reduced enzyme activity and slower clearance of ibuprofen from the body, increasing the risk of ibuprofen-related ADR [5, 6]. This is the first report addressing the association between combined *CYP2C9*∗*3 and CYP2C8*∗*3* variant alleles with ibuprofen-induced upper GI toxicity in a Saudi patient.

The frequencies of the *CYP2C9*∗*3* allele have been extensively studied and are known to vary significantly across different populations, as reported by the 1000 Genome Phase 3 project [23]. The European population exhibits a high frequency of the *CYP2C9*∗*3* allele (11%), whereas its occurrence is rare in America (4%) and East Asia (3%) and absent in Africa. Conversely, South Asian populations, such as Pakistan (11.9%) and Bangladesh (11.6%), show a remarkably high prevalence of the *CYP2C9*∗*3* allele. In Western Saudi populations, the presence of the *CYP2C9*∗*3* allele reaches 54%, which is considerably higher than the reported frequencies in other populations (ranging from 0% to 11%) [24]. Regarding the *CYP2C8*∗*3* allele, it emerges as the most prevalent variant allele among Europeans (11.2%) and Americans (6.7%). Similarly, in Western Saudi populations, the *CYP2C8*∗*3* allele frequency is approximately 8.57%, accounting for 50.02% of all variant *CYP2C8* alleles in Saudis [25]. In contrast, South Asians (<4%), Africans (2.1%), and East Asians (<1%) exhibit significantly lower frequencies of the *CYP2C8*∗*3* allele. Given the notable prevalence of *CYP2C9*∗*3* and *CYP2C8*∗*3* variant alleles within the Saudi population, there is a potential concern regarding an increased susceptibility of Saudis to experience ADRs specifically associated with drugs metabolized by CYP2C9 and CYP2C8 enzymes, including ibuprofen.

A previous study reported that subjects who were double-heterozygous or homozygous for the *CYP2C9*∗*3* and *CYP2C8*∗*3* alleles had significantly low clearance rates for ibuprofen, ranging between 7% and 27% of the average clearance rate for subjects carrying the wild-type alleles [19]. The low clearance rate of individuals carrying the *CYP2C9*∗*3* and *CYP2C8*∗*3* variant alleles resulted in an increase in ibuprofen plasma concentrations and half-life. Therefore, toxicity may occur more frequently or be more severe at higher plasma concentrations. In patients using ibuprofen, *CYP2C9*∗*3* and *CYP2C8*∗*3* variant alleles significantly predict the likelihood of developing GI bleeding [26]. Our patient's genetic test results revealed that the patient was homozygous for both *CYP2C9*∗*3* and *CYP2C8*∗*3* variant alleles. Therefore, the patient was considered a poor metabolizer of both the CYP2C9 and CYP2C8 enzymes, suggesting that the patient had a poor metabolism of ibuprofen.

In the case of the patient under investigation, ibuprofen was administered at a dosage of 600 mg, resulting in a total daily dose of 1200 mg, which is equal to the established Defined Daily Dose (DDD) for ibuprofen as designated by the World Health Organization [27]. Recent research conducted by Forgerini et al. has demonstrated an increased risk of upper GI bleeding in individuals who carry the *CYP2C9*∗*3* allele and are taking more than 0.5 DDDs of NSAIDs [28]. Considering that our patient was a carrier of the *CYP2C9*∗*3* allele and utilized ibuprofen at a dose more than 0.5 DDD, it indicates an augmented vulnerability to upper GI bleeding at administrated dosage. This finding may imply the existence of a gene-dose effect, where individuals with the *CYP2C9*∗*3* allele are more likely to experience elevated risk and severity of NSAID-induced ADRs.

According to the CPIC guideline concerning CYP2C9 enzyme and NSAIDs, individuals identified as poor metabolizers for CYP2C9 should initiate ibuprofen therapy at a dosage ranging from 25% to 50% of the recommended initial dose [29]. Careful titration of the dose is advised to attain a clinical response and reach a dose between 25% and 50% of the recommended amount. To align with each patient's treatment objectives, it is recommended to administer ibuprofen for the shortest possible duration using the lowest effective dose. For CYP2C9 poor metabolizers, it is further recommended to consider prescribing alternative NSAIDs that do not undergo metabolism *via* the CYP2C9 enzyme, such as naproxen, sulindac, aspirin, or other pain management strategies [29]. Furthermore, caution is warranted when prescribing ibuprofen to patients carrying the *CYP2C8*∗*3* variant allele since the CYP2C8 enzyme also participates in the metabolism of ibuprofen. CPIC recommends a lower ibuprofen dose for CYP2C8 poor metabolizers. While the *CYP2C8*∗*3* allele alone may not significantly reduce ibuprofen clearance, it is in strong linkage disequilibrium with the *CYP2C9*∗*2* allele, resulting in impaired ibuprofen metabolism and elevated plasma levels of the drug [30]. Other genetic and clinical factors may also influence ibuprofen metabolism [29]. The choice of NSAID should be based on the patient's treatment goals and toxicity risks.

The patient enrolled in our study has been undergoing methotrexate treatment for approximately one year as part of her RA management. Notably, over the past eight weeks, she has been self-administering ibuprofen without a prescribed regimen. Therefore, it is crucial to acknowledge the potential risks associated with the simultaneous use of methotrexate and NSAIDs in patients with RA. Multiple studies have consistently demonstrated that the concomitant use of methotrexate and NSAIDs can substantially increase the likelihood of experiencing severe ADRs. These ADRs encompass GI adverse effects, liver toxicity, acute renal failure, and cytopenia [31]. Notably, NSAIDs have been shown to elevate methotrexate levels in the blood, thereby amplifying the associated side effects of methotrexate therapy. A recent case report by Tun et al. documented a rare methotrexate-induced gastric ulcer. This case highlights the potential but rare GI complications arising from methotrexate use [32]. Taking into consideration the patient's concomitant use of methotrexate and NSAIDs, the upper GI bleeding observed could potentially be attributed to a drug-drug interaction. These findings emphasize the importance of exercising caution and implementing vigilant monitoring when managing patients who are prescribed both methotrexate and NSAIDs concurrently.

## 4. Conclusion

Ibuprofen, a widely utilized NSAID for RA management, has been associated with potential ADRs, including GI ulcers and bleeding. These risks can be influenced by genetic variations in the *CYP2C9* and *CYP2C8* genes. Although the CPIC does not recommend routine screening for *CYP2C9* and *CYP2C8* non-functional alleles before ibuprofen therapy, a comprehensive understanding of these genetic variations can assist in optimizing ibuprofen dosage and reducing the risk of ADRs in RA patients. Therefore, incorporating genetic testing into the clinical decision-making process for NSAID treatment in RA patients has significant implications. It enables personalized medicine approaches, optimizes dosage, minimizes ADRs, and facilitates long-term treatment planning [33].

## Data Availability

The data used to support the findings of this study are included within the article.
